# Supplementary material for: Sero-Epidemiological Study of Bordetella pertussis Infection in the Italian General Population
Source: Vaccines (Basel). 2022 Dec 13;10(12):2130. doi: 10.3390/vaccines10122130 (PMC9782223; doi:10.3390/vaccines10122130)
Supplement: Supplementary file 1 [file vaccines-10-02130-s001.zip › vaccines-1991004-SI.pdf]

vaccines

Sero-epidemiological Study Group

Bordino V [1], Cornio A[1], Greco F [2], Giandomenico A [2], Montomoli E [3], Trombetta CM [3], Baldo V [4], Baldovin T [4], De Fino M [5], Marandola M [6], Orsi A [7], Ricucci V [8], Barrocci S [9], La Porta R [9], Raspa P [10], Castiglia P [11], Dettori M [11], Ghisellini S [12], Boni M [12], Tramuto F [13], Robatscher E [14], Gamper A [14], Panico A[15], Angelillo IF [16], Napolitano F [16], De Motoli F [17], Flacco ME [18]

[1] Dept. of Sciences of Public Health and Pediatrics, University of Turin, Italy; [2] Unit of Microbiology and Virology, "Annunziata" Hospital, Cosenza, Italy; [3] Dept. of Molecular and Developmental Medicine, University of Siena, Italy; [4] Dept. of Cardiac, Thoracic, Vascular Sciences and Public Health, Unit of Hygiene and Public Health, University of Padova, Italy; [5] General Manager of Umbria2 LHA, Terni, Italy, former Manager of Healthcare District of Lauria, Potenza, Italy; [6] Unit of Hygiene and Public Health, Potenza LHA, Italy; [7] Dept. of Health Sciences, University of Genova, Italy; [8] Unit of Hygiene, IRCCS San Martino Hospital, Genova, Italy; [9] Unit of Clinical Pathology, Area Vasta 1, Clinical Laboratory of "S. Maria della Misericordia" Hospital, Urbino, Italy; [10] Clinical Laboratory, Ozieri, ATS Sardegna, Sassari LHA, Italy; [11] Department of Medicine, Surgery and Pharmacy, University of Sassari, Italy; [12] UOC Provincial Laboratory, University Hospital of Ferrara, Italy; [13] Dept. PROMISE, University of Palermo; [14] Microbiology and Virology Laboratory, Health District of Bolzano, Alto Adige Health Authority, Italy; [15] Dept. of Biological and Environmental Sciences and Technologies, University of Salento, Lecce, [16] Dept. of Experimental Medicine, University of Campania "Luigi Vanvitelli", Naples, Italy, [17] Post-graduate School of Hygiene and Preventive Medicine, University of Ferrara, Ferrara, Italy, [18] Dept. of Environment and Prevention Sciences, University of Ferrara, Ferrara, Italy
